# Neoadjuvant FinHer regimen in patients with HER2-positive breast cancer: a retrospective audit

**DOI:** 10.3389/fonc.2026.1752548

**Published:** 2026-05-18

**Authors:** Anoop T. M., Rona Joseph, Sherin Mathew, Mintu Mathew, Saikumar Soman, Kalai Bharathi, Senthamizhan S

**Affiliations:** 1 Department of Medical Oncology, Regional Cancer Centre, Thiruvananthapuram, Kerala, India; 2 Department of Medical Oncology, PRS Hospital, Thiruvananthapuram, Kerala, India; 3 Department of Medical Oncology, Bagchi Sri Shankara Cancer Centre and Research Institute, Bhubaneswar, India

**Keywords:** FinHer protocol, HER2-positive breast cancer, neoadjuvant, pCR, trastuzumab

## Abstract

**Introduction:**

The role of trastuzumab in the neoadjuvant setting has been established by multiple large randomized trials. However, there are limited data on the efficacy and survival outcomes of the FinHer regimen in the neoadjuvant setting for the treatment of HER2-positive breast cancer in the South Indian population.

**Methods:**

This retrospective audit included patients with locally advanced HER2-positive breast cancer who received neoadjuvant chemotherapy with a short course of trastuzumab (nine weekly doses) following the FinHer protocol in a resource-limited setting. The primary endpoint of the study was pathological complete response (pCR). Secondary endpoints included disease-free survival (DFS) and overall survival (OS) at 5 years. The primary study population comprised stage III patients, while patients with oligometastatic disease were included as an exploratory cohort.

**Results:**

This retrospective audit included 100 patients. Of these, 76 patients had stage III disease and 24 had oligometastatic disease. The pCR rate was 30% following the neoadjuvant FinHer protocol. Estrogen receptor (ER) and progesterone receptor (PR) positivity were associated with decreased pCR rates, which were statistically significant (*p* = 0.002). Patients with the HER2-enriched subtype (47%) achieved higher pCR rates compared to those with the luminal B HER2-positive subtype (21.2%), with a statistically significant *p*-value (*p* = 0.008). On univariate and multivariate analyses, stage was the only significant predictor of DFS (*p* = 0.003) and OS (*p* = 0.002). Stage III disease remained independently associated with improved survival outcomes compared to oligometastatic disease [OS: hazard ratio (HR) 0.35, 95% confidence interval (CI) 0.18–0.70, *p* = 0.003; DFS: HR 0.40, 95% CI 0.21–0.76, *p* = 0.005]. Achievement of pCR was associated with a numerically lower risk of death and recurrence (OS: HR 0.56, 95% CI 0.24–1.30; DFS: HR 0.51, 95% CI 0.22–1.16).

**Conclusion:**

The neoadjuvant FinHer protocol resulted in a pCR rate of 30% in locally advanced HER2-positive breast cancer. ER/PR positivity was associated with decreased pCR rates. Achievement of pCR was associated with a numerically lower risk of death and recurrence. Stage III disease remained independently associated with improved survival outcomes compared to oligometastatic disease.

## Introduction

The advent of trastuzumab has revolutionized the treatment of breast cancer, and it is currently used in the neoadjuvant, adjuvant, and metastatic settings for patients with HER2-positive breast cancer (1). The recommended duration of trastuzumab therapy in breast cancer treatment, whether in the adjuvant or neoadjuvant setting, is a total of 17 cycles. However, several trials have explored the use of trastuzumab in abbreviated regimens (2–4). The FinHer trial paved the way for multiple studies evaluating chemotherapy combined with shorter courses of trastuzumab, with conflicting results regarding survival compared to longer durations of trastuzumab treatment (5). Meta-analyses have suggested that shorter courses of trastuzumab may be an alternative for patients with lower-risk disease and for those with cardiac dysfunction (6). Shorter courses of trastuzumab may be an attractive option for reducing the financial burden of treatment, decreasing trastuzumab-related toxicities, and significantly shortening the duration of therapy, particularly in middle- and low-income countries in Asia. At our center, many patients were unable to afford 1 year of adjuvant trastuzumab during that period. Consequently, the FinHer protocol was adopted for patients with HER2-positive breast cancer in both adjuvant and neoadjuvant settings. The role of trastuzumab in the neoadjuvant setting has been established by multiple large randomized trials (7–11). However, there is limited evidence regarding the efficacy and survival outcomes of shorter courses of trastuzumab in the neoadjuvant treatment of HER2-positive breast cancer. The clinico-epidemiological pattern of HER2-positive breast cancer in Asian populations differs from that in Western populations, with younger age at presentation and more advanced-stage disease being more common (12). This places a substantial financial burden on both families and national healthcare systems. Therefore, the aim of this study was to evaluate the effectiveness of the FinHer protocol as a neoadjuvant treatment in terms of pathological response and 5-year survival outcomes among patients with HER2-positive breast cancer in a resource-limited setting.

## Methods

This study was a single-center, retrospective audit of consecutively treated patients with HER2-positive locally advanced breast cancer who received neoadjuvant chemotherapy with a short course of nine weekly trastuzumab cycles (FinHer protocol) at the Breast Clinic, Department of Medical Oncology, Regional Cancer Centre, Thiruvananthapuram, between 1 January 2017 and 30 December 2018. This research audit was approved by the Institutional Review Board (IRB No: 12/2019/02).

The inclusion criteria comprised all newly diagnosed, consecutively treated patients with locally advanced HER2-positive breast cancer who received neoadjuvant chemotherapy with a short course of nine weekly trastuzumab cycles. Patients aged >14 years and <70 years were included. Only stage III patients and those with oligometastatic disease were eligible. The primary study population consisted of stage III patients, while patients with oligometastatic disease were included for exploratory analysis. Patients with stage I and II disease, including those classified as high risk, were excluded. The exclusion of high-risk patients was based on tumor board decisions, as the study primarily focused on locally advanced breast cancer in the neoadjuvant setting. Patients with ≤5 metastases in total and metastases measuring ≤5 cm, amenable to local therapy, were classified as having oligometastatic breast cancer. The number and size of metastases were confirmed using whole-body CT (computed tomography), FDG-PET/CT (positron emission tomography/CT), or MRI (magnetic resonance imaging). All oligometastatic lesions identified on radiological imaging were discussed and reviewed by an institutional radiologist. The ER/PR status and human epidermal growth factor receptor 2 (HER2/neu) status were determined by immunohistochemistry (IHC). In cases where HER2-positive results were equivocal on IHC, fluorescence *in situ* hybridization (FISH) was performed. HER2 positivity was defined as IHC 3+ or IHC 2+ with positive amplification on FISH. Traditional immunohistochemical biomarkers (ER, PR, HER2, and Ki-67 expression) were used to classify tumors into molecular subtypes. Luminal B (HER2-positive) subtype was defined as ER-positive with PR either positive or negative, and HER2-positive. The HER2-positive/HR-negative tumor subtype was defined as ER-negative, PR-negative, with HER2 amplification or overexpression. Gene expression profiling was not performed due to its unavailability at our center.

Exclusion criteria included patients who had previously received chemotherapy, those treated with palliative intent, patients with relapsed or previously treated HER2-positive breast cancer, and patients with cardiovascular, liver, or pulmonary disease who were unfit for trastuzumab and chemotherapy. All patients underwent pre-NACT evaluation, including a complete medical history, physical examination, baseline blood tests, and imaging studies such as bone scintigraphy, CT scans, MRI, or PET-CT, as per institutional practice. Monitoring was conducted through clinical examination at every visit and imaging after completion of chemotherapy or when clinically indicated. Radiological response to treatment in solid tumors was assessed using the Response Evaluation Criteria in Solid Tumors (RECIST), version 1.1. Out of 1,200 patients diagnosed with breast carcinoma during the study period, only 100 patients with HER2-positive locally advanced breast cancer received the neoadjuvant FinHer protocol. A baseline cardiac clinical examination and echocardiography were performed as part of routine pre-chemotherapy evaluation. Echocardiographic assessments were conducted at baseline before initiating weekly trastuzumab and again before starting FEC therapy. Only patients with adequate cardiac function, defined as a normal LVEF ≥50% on echocardiography, were included. Patients with a documented history of congestive heart failure, myocardial infarction, angina requiring medication, uncontrolled hypertension, clinically significant valvular disease, or unstable arrhythmias were excluded. However, patients with controlled hypertension on medication and diabetes were included. Cardiotoxicity was defined as a decrease in LVEF below 50%, an absolute reduction of more than 10 percentage points from baseline, or the presence of signs or symptoms of heart failure.

Those patients with an asymptomatic drop in EF were serially monitored after withholding trastuzumab and were started on angiotensin receptor blockers (ARBs). Once their EF returned to baseline values, they were rechallenged. Patients with symptomatic cardiac toxicity were not rechallenged. All patients were advised to continue ARB therapy for 6 months after completion of treatment. These patients were monitored for EF loss by echocardiography every month for the first 3 months, every 3 months for the next 6 months, and every 6 months thereafter.

### Treatment protocol

Patients received nine weekly intravenous infusions of trastuzumab in combination with docetaxel. Trastuzumab was administered at a dose of 4 mg/kg on day 1 over 90 min, followed by 2 mg/kg weekly thereafter, administered over 30 min. Trastuzumab was given prior to docetaxel, with the first infusion administered on day 1 of the first docetaxel cycle. Docetaxel was administered intravenously every 21 days for a total of three cycles. This was followed by three cycles of 5-fluorouracil (500 mg/m²), epirubicin (90 mg/m²), and cyclophosphamide (500 mg/m²) (FEC), administered every 21 days. Additional supportive care was provided as required. Surgery was performed 3 to 4 weeks after completion of neoadjuvant therapy.

As per institutional practice, all patients with stage III disease and oligometastatic disease who were intended for radical treatment after NACT underwent either modified radical mastectomy (MRM) or breast-conserving surgery (BCS). A standardized institutional pathological assessment was performed. Radiation and hormonal therapy were administered according to standard guidelines after completion of the treatment protocol. Patients who were estrogen receptor-positive or progesterone receptor–positive received adjuvant endocrine therapy, such as aromatase inhibitors or tamoxifen, based on menopausal status. All patients who received neoadjuvant therapy were given adjuvant postmastectomy radiation. Post-treatment follow-up was conducted as per institutional practice. Regular follow-up visits were scheduled every 3 months during the first 3 years following treatment, every 6 months from years 4 to 5, and annually thereafter. A physical examination and ultrasound of the abdomen and pelvis were performed every 3 months. CT of the chest, abdomen, and pelvis was performed every 6 months, and a mammogram was performed annually.

### Endpoints and definitions

The primary endpoint of the study was the pathological complete response (pCR) rate. The secondary endpoint was OS. pCR was defined as the absence of residual invasive disease in the breast and axilla (ypT0/is, ypN0). This definition allows for the presence of ductal carcinoma *in situ* (DCIS) in the breast but requires no invasive cancer in the breast and no cancer in the lymph nodes.

OS was calculated from the date of completion of treatment to death from any cause.

The date of the last routine follow-up was used for survival analysis. Patients who missed their scheduled follow-up were contacted telephonically to assess their current status.

### Statistical methods

Descriptive statistics were presented as frequencies and percentages for categorical variables, and as mean or median with standard deviation for continuous variables. The statistical significance of categorical variables was assessed using the chi-square test. Progression-free survival was estimated using the Kaplan–Meier method. Survival curves generated using the Kaplan–Meier method were compared using the log-rank test. A *p*-value < 0.05 was considered statistically significant.

In addition to univariable analyses, a multivariable Cox proportional hazards model was constructed to evaluate factors associated with OS and DFS. Clinically relevant variables, including stage group (stage III vs. oligometastatic), pCR, and hormone receptor status (ER/PR positive vs. negative), were included in the model. However, hormone receptor status could not be reliably estimated in the multivariable model due to limited variability within the dataset. Hazard ratios (HRs) with 95% confidence intervals (CIs) were reported. Proportional hazards assumptions were assessed using Schoenfeld residuals.

## Results

### Baseline characteristics

**Table 1** describes the baseline characteristics of patients enrolled in this study population. A total of 100 patients were included in the study. The median age at presentation was 53 years. The major comorbidities were hypertension (24%), diabetes mellitus (33%), and cardiovascular disease (3%). The majority of patients were postmenopausal (68%), followed by premenopausal (23%) and perimenopausal (9%). The stage at presentation was stage III in 76% of patients and oligometastatic disease in 24%. Approximately 66% of patients had luminal B HER2-positive breast cancer, while 34% had HER2-enriched breast cancer. Tumor grade was grade I in 2%, grade II in 32%, and grade III in 66% of patients. Eighty-eight percent of patients underwent MRM, while 12% underwent BCS. All patients received adjuvant radiotherapy. All patients with oligometastatic disease (24%) underwent MRM, and none underwent BCS. Ninety-two percent of patients received 40 Gy in 15 fractions, whereas 8% received 50 Gy in 25 fractions. Sixty-six patients received adjuvant hormonal therapy; among them, 14 received tamoxifen and 52 received aromatase inhibitors. Eighteen patients received zoledronic acid infusions for bone metastasis. The treatment-related toxicities observed in our study are summarized in **Table 1**. The most common toxicity was hematological, followed by hepatotoxicity, mucositis, pulmonary toxicity, diarrhea, and cardiac toxicity. Among all toxicity grades, 38% of patients experienced grade I toxicity, while 14% experienced grade II toxicity. Five percent of patients developed grade III toxicity. None of the patients experienced grade IV toxicity. Among those with grade III toxicity, three patients had grade III mucositis, and two patients developed grade III pneumonitis secondary to docetaxel. Dose reduction was implemented for the FEC regimen due to grade III mucositis. Docetaxel was discontinued in patients who developed grade III pneumonitis. Pneumonitis was managed with steroids and supportive care. In the overall study population, 66 patients had luminal B HER2-positive disease and received adjuvant hormonal treatment. Seventeen patients (17%) experienced cardiac toxicity in the form of asymptomatic LVEF decline. Only two patients developed symptomatic LVEF decline. In all cases, LVEF returned to baseline after discontinuation of trastuzumab for 2 weeks. All patients were started on ACE inhibitors upon diagnosis of asymptomatic LVEF decline. None of the patients experienced cardiac events.

**Table 1:** Baseline characteristics.

Study cohort	*N* = 100
Median age, years (range)	53 (32–73)
Comorbidities, *n* (%)	
Hypertension	24
Diabetes mellitus	33
Coronary artery disease	3
Menopausal status, *n* (%)	
Premenopausal	23
Postmenopausal	68
Perimenopausal	9
Stage, *n* (%)	
Stage III	76
Oligometastatic disease	24
Grade, *n* (%)	
I	0
II	34
III	66
Molecular type, *n* (%)	
Luminal B HER2-positive	66
HER2-enriched	34
Toxicities, *n* (%)	
Hematological toxicities	48
Cardiotoxicity	17
Asymptomatic LVEF loss	
Hepatotoxicity	6
Mucositis	5
Diarrhea	3
Pulmonary toxicity	4
Highest grade of toxicity, *n* (%)	
Grade I	38
Grade II	14
Grade III	5
Surgery, *n* (%)	
Modified radical mastectomy (MRM)	88
Breast conservation surgery (BCS)	12
Complete pathological response (pCR), *n* (%)	
pCR	30
No pCR	70
Radiation, *n* (%)	
Adjuvant radiotherapy	100
40 Gy/15#	92
50 Gy/25#	8
**Relapse, *n* (%)**	42
Site of relapse, *n* (%)	
Local	12
Opposite breast	1
Liver	5
Bone	8
Lung	7
Brain	8
Survival	
5-year DFS	57.8% ± 5.1%
5-year OS	61.6% ± 5%

Bold values indicate statistically significant p-values.

### Pathological complete response

pCR was achieved in 30 patients (30%) following the neoadjuvant FinHer protocol, while 70% of patients did not achieve pCR. Breast-only pCR was observed in 38% of patients, axilla-only pCR in 55%, and combined breast and axilla pCR in 30% of patients. Positivity was associated with decreased pCR rates, which was statistically significant (*p* = 0.002). Patients with the HER2-enriched subtype (47%) achieved higher pCR rates compared to those with the luminal B HER2-positive subtype (21.2%), with a statistically significant *p*-value of 0.008 (**Table 2**). None of the variables, including age, menopausal status, or tumor grade, showed a significant association with pCR in patients treated with the FinHer protocol. None of the patients who did not achieve pCR received adjuvant treatment such as T-DM1 due to financial constraints. The most common sites of oligometastasis were bone (18%), lung nodules (4%), and liver (2%). Similarly, the most common sites of metastasis were bone (18%), lung nodules (4%), and liver (2%).

**Table 2:** Association of PCR with clinico-pathological variables.

Variables	PCR	No PCR	*p*-value (chi-square)
Age <65 years	25	63	0.347
Age >65 years	5	7	
Premenopausal and perimenopausal	8	24	0.454
Postmenopausal	22	46	
Stage III	24	52	0.540
Stage IV (oligometastatic)	6	18	
Luminal B HER2-	14	52	**0.008**
positive	16	18	
HER2-enriched			
Grade II	12	22	0.407
Grade III	18	48	

Bold values indicate statistically significant p-values.

### Recurrence

At a median follow-up of 46 months (range, 31–60 months), 42 patients (42%) developed recurrence. The site of recurrence was the local chest wall in 12% of patients and the contralateral breast in 1%. Systemic recurrence was observed in 28% of patients, including the brain (8%), bone (8%), lung (7%), and liver (5%). Recurrences were treated with systemic chemotherapy plus trastuzumab in 12% of patients and with a combination of radiotherapy/surgery and chemotherapy plus trastuzumab in 29% of patients. Among the 100 patients, 37 (37%) had died by the last follow-up in April 2023. No patients were lost to follow-up.

#### Survival of stage III patients

A total of 76 patients with stage III disease were included in this study, excluding 24 patients with oligometastatic disease.

**Figure 1:**
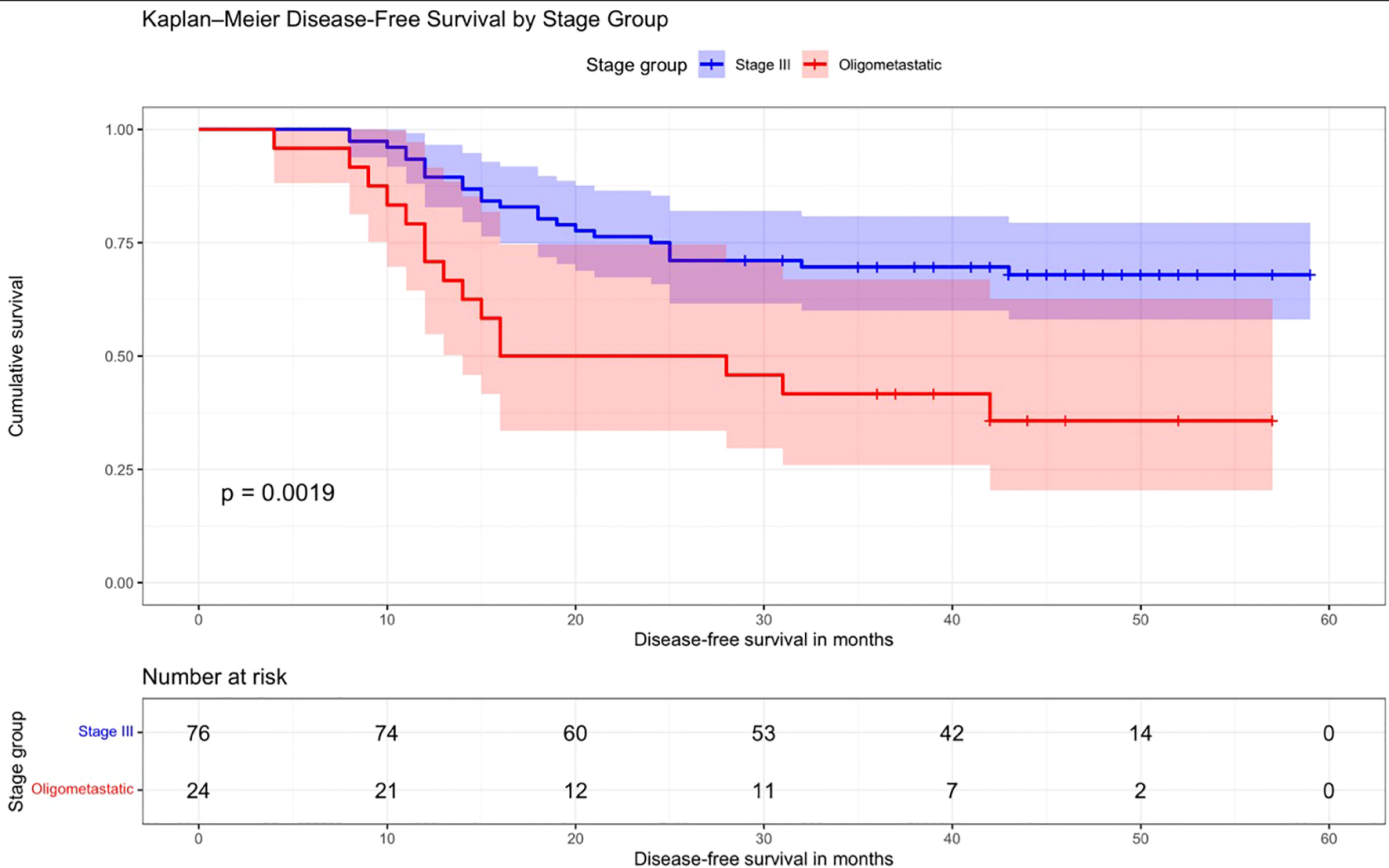
Disease-free survival in patients with stage III and oligometastatic disease. Kaplan–Meier curve comparing disease-free survival (DFS) between patients with stage III disease and those with oligometastatic disease. Disease-free survival was significantly better in stage III patients than in the oligometastatic group. The 5-year DFS was approximately 67% in stage III patients versus 36% in the oligometastatic group (*p* = 0.0019).

The Kaplan–Meier curve comparing DFS between patients with stage III disease and those with oligometastatic disease is presented in **Figure 1**. The analysis showed significantly better DFS in stage III patients compared to the oligometastatic group. The 5-year DFS was approximately 67% in stage III patients versus 36% in the oligometastatic group (*p* = 0.0019) (**Figure 1**).

**Figure 2:**
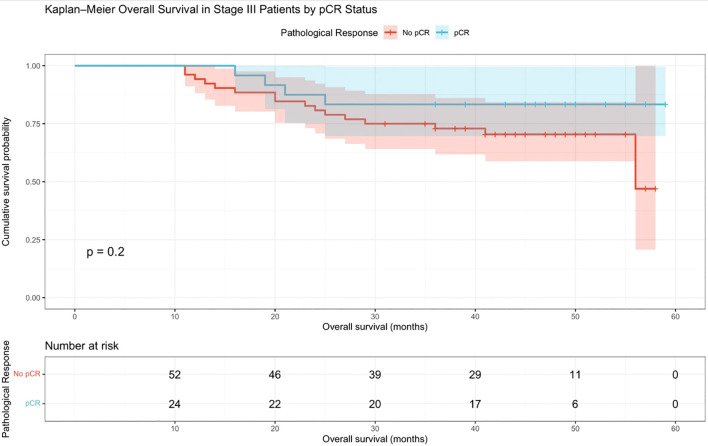
Overall survival in patients with stage III and oligometastatic disease. Kaplan–Meier curve comparing overall survival (OS) between patients with stage III disease and those with oligometastatic disease. OS was significantly better in stage III patients than in the oligometastatic group. The 5-year OS was approximately 60% in stage III patients versus 26% in the oligometastatic group (*p* = 0.0011).

A Kaplan–Meier curve demonstrating the comparison of OS between patients with stage III disease and those with oligometastatic disease is shown in **Figure 2**. It demonstrates significantly better OS in stage III patients compared to the oligometastatic group. The 5-year OS was approximately 60% in stage III patients versus 26% in the oligometastatic group (p = 0.0011) (**Figure 2**).

**Figure 3:**
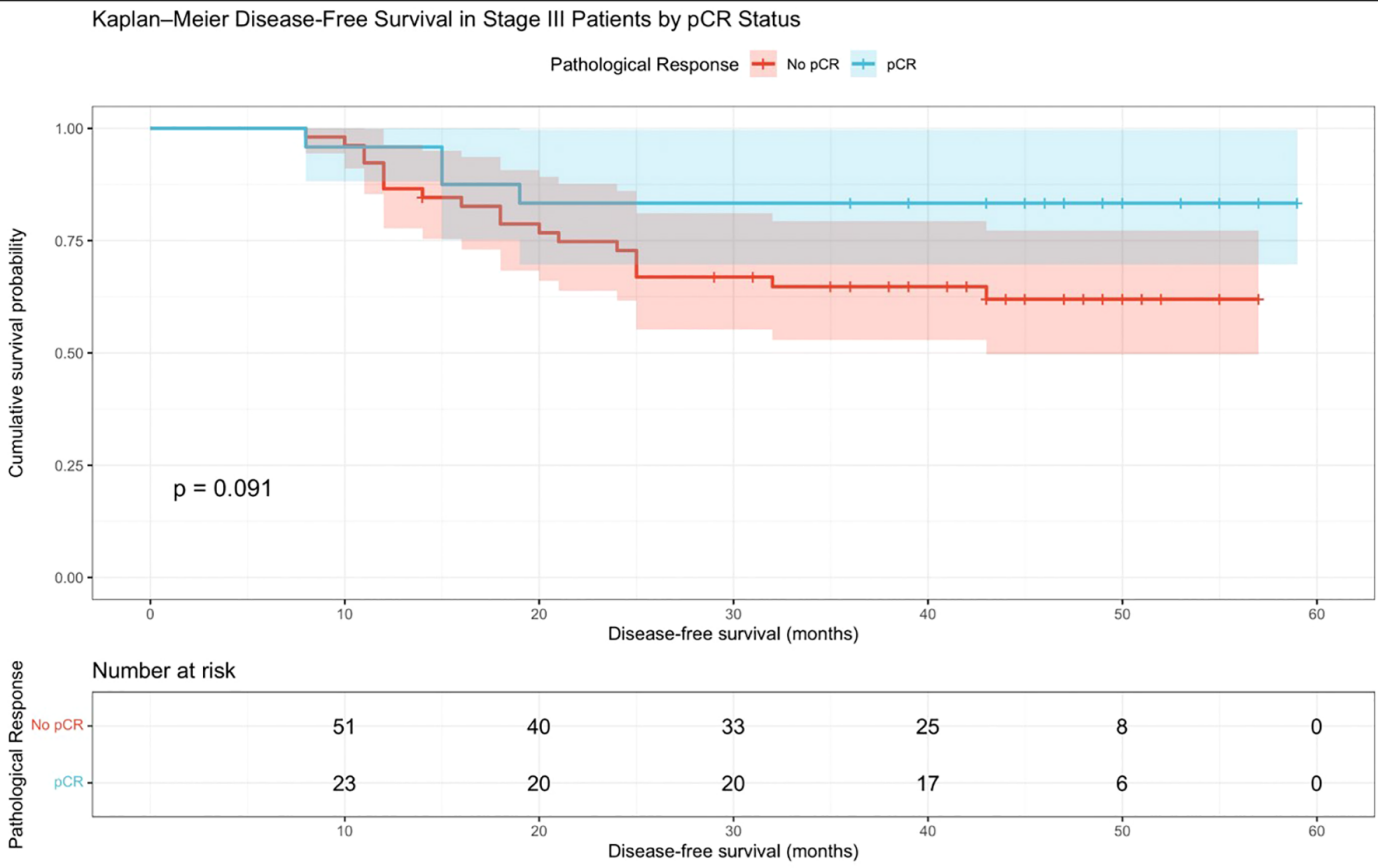
DFS in stage III patients with pCR versus no pCR. Kaplan–Meier curve showing disease-free survival (DFS) among stage III patients stratified by pCR status. Patients who achieved pCR demonstrated numerically better DFS than those without pCR; however, the difference did not reach statistical significance (log-rank *p* = 0.091). At baseline, the numbers at risk were 52 in the no-pCR group and 24 in the pCR group. At approximately 50 months, the numbers at risk were 8 and 6, respectively. The estimated 5-year DFS was higher in the pCR group (approximately 83%–84%) than in the no-pCR group (approximately 62%).

A Kaplan–Meier curve showing DFS among stage III patients stratified by pCR versus no pCR is shown in **Figure 3**. Patients who achieved pCR demonstrated numerically better DFS compared to those without pCR; however, the difference did not reach statistical significance (log-rank *p* = 0.091). At baseline, the numbers at risk were 52 in the no-pCR group and 24 in the pCR group. At approximately 50 months, the numbers at risk were 8 and 6, respectively. The estimated DFS at approximately 5 years was visually higher in the pCR group (approximately 83%–84%) than in the no-pCR group (approximately 62%).

**Figure 4:**
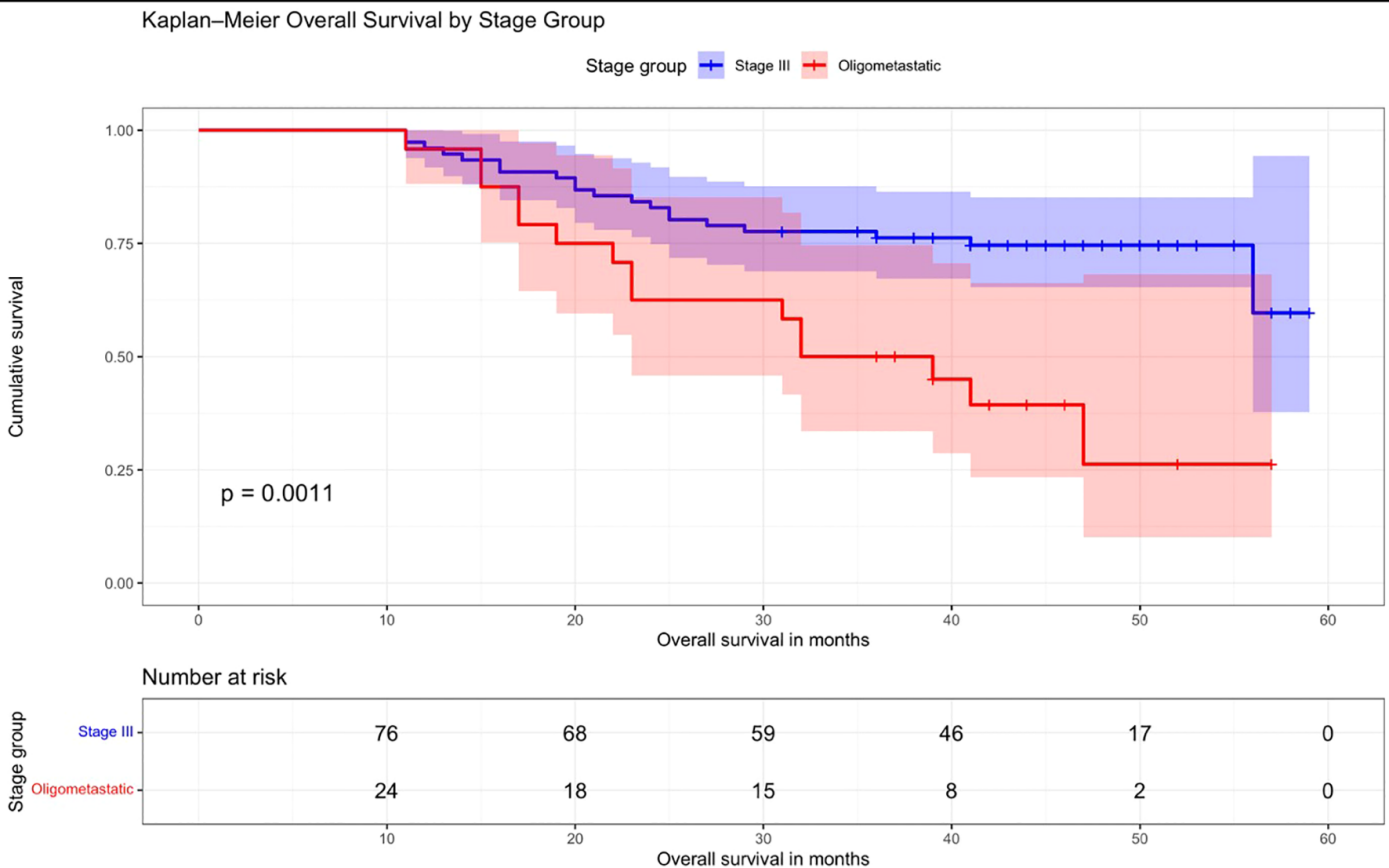
OS in stage III patients with pCR versus no pCR. Kaplan–Meier curve showing overall survival (OS) among stage III patients stratified by pCR status. Patients who achieved pCR demonstrated numerically better OS than those without pCR; however, the difference was not statistically significant (log-rank *p* = 0.20). At baseline, the numbers at risk were 51 in the no-pCR group and 23 in the pCR group. At approximately 50 months, the numbers at risk were 11 and 6, respectively. The estimated 5-year OS was higher in the pCR group (approximately 84%) compared with the no-pCR group (approximately 47%).

The Kaplan–Meier curve showing OS among stage III patients stratified by pCR versus no pCR is presented in **Figure 4**. Patients who achieved pCR demonstrated numerically better OS than those without pCR; however, this difference was not statistically significant (log-rank *p* = 0.20). At baseline, the numbers at risk were 51 in the no-pCR group and 23 in the pCR group. At approximately 50 months, the numbers at risk were 11 and 6, respectively. The estimated 5-year OS appeared higher in the pCR group (approximately 84%) compared with the no-pCR group (approximately 47%).

Among stage III patients, 24 achieved pCR, whereas 52 did not. Of the 52 patients without pCR, 19 experienced recurrence, compared with 4 recurrences among the 24 patients who achieved pCR. The median DFS was 43 months, with a median follow-up of 54 months. There was no statistically significant association between pCR and disease recurrence in stage III patients (*p* = 0.091) (**Supplementary Figure 1**). The median OS for stage III patients was 49 months, with a median follow-up of 54 months. Among the 76 patients, there were 4 deaths in the pCR group and 16 deaths in the no-pCR group. There was no statistically significant association between pCR and mortality (*p* = 0.197) (**Supplementary Figure 2**).

Among the 76 stage III patients, 25 were ER/PR-negative, of whom 13 achieved pCR. In contrast, among the 51 ER/PR-positive patients, only 11 achieved pCR. There was a statistically significant association between ER/PR negativity and achievement of pCR (*p* = 0.007).

#### Survival of oligometastatic disease

Twenty-four patients included in the study had oligometastatic disease at presentation but were treated with radical intent. These patients had a median DFS of 16 months and a median OS of 32 months. Among the 24 patients with oligometastatic disease, 6 achieved pCR, while 18 did not. Approximately 15 patients with luminal B HER2-positive disease in the oligometastatic group received adjuvant hormonal therapy. All patients with oligometastatic disease who underwent NACT experienced relapse by the last data cutoff.

### Cox regression analysis

On univariate analysis, stage was the only significant predictor of DFS (*p* = 0.003) and OS (*p* = 0.002) (**Table 3**). None of the other factors—age, menopausal status, tumor grade, pathologic complete response (pCR vs. no pCR), type of surgery (BCS vs. MRM), or molecular subtype (luminal B HER2-positive vs. HER2-enriched)—were significantly associated with DFS or OS.

**Table 3:** Univariate analysis of OS and DFS.

Variables	OS univariate analysis			DFS univariate analysis		
	Hazard ratio	Confidence interval	*p*-value	Hazard ratio	Confidence interval	*p*-value
Age <65 years	1		0.737	1		0.219
Age >65 years	0.841	0.298–2.374		0.479	0.148–	
					1.549	
Premenopausal and perimenopausal	1		0.242	1		0.082
	0.668	0.34–1.29		0.580	0.314–	
Postmenopausal					1.071	
Stage III	1			1		
Stage IV oligometastatic	2.916	1.5–5.65	**0.002**	2.623	1.38–4.9	**0.003**
Luminal B HER-2	1		0.924	1		0.815
positive	1.033	0.526–2.03		1.079	0.568–	
HER2-enriched					2.051	
Grade II	1		0.382	1		0.110
Grade III	1.34	0.69–2.59		1.64	0.89–3.03	
PCR	1		0.143	1		0.109
No PCR	1.746	0.79–3.82		1.83	0.874–3.84	
BCS	1		0.737	1		0.39
MRM	2.91	0.7–12.1		1.5	0.5–4.31	

Bold values indicate statistically significant p-values.

Multivariable Cox regression analysis, including stage group, pathologic complete response (pCR), and hormone receptor status, was performed for OS and DFS (**Table 4**). Stage III disease remained independently associated with improved survival outcomes compared with oligometastatic disease (OS: HR 0.35, 95% CI 0.18–0.70, *p* = 0.003; DFS: HR 0.40, 95% CI 0.21–0.76, *p* = 0.005). Achievement of pCR was associated with a numerically lower risk of death and recurrence (OS: HR 0.56, 95% CI 0.24–1.30; DFS: HR 0.51, 95% CI 0.22–1.16), although these differences did not reach statistical significance. Hormone receptor status was included in the multivariable model; however, its effect could not be estimated reliably, as the model yielded a non-estimable coefficient (NA), likely due to limited variability in the dataset with an imbalance between hormone receptor–positive and -negative patients (51 vs. 25) and a small number of events within subgroups, resulting in model instability. This likely reflects limited statistical power rather than absence of biological significance.

**Table 4:** Multivariable Cox regression analysis for OS and DFS.

Variable	Overall survival HR (95% CI)	*p*-value	Disease-free survival HR (95% CI)	*p*-value
Stage III vs. oligometastatic	**0.35 (0.18–0.70)**	**0.003**	**0.40 (0.21–0.76)**	**0.005**
pCR vs. no pCR	0.56 (0.24–1.30)	0.18	0.51 (0.22–1.16)	0.11

Bold values indicate statistically significant p-values.

### Survival of the combined population

Five-year DFS and OS for the total study population, stratified by pCR status, are shown in **Supplementary Figures 1** and **2**. The Kaplan–Meier curve comparing 5-year DFS between patients achieving pCR and those without pCR in the overall study population is presented in **Supplementary Figure 3**. It demonstrates improved DFS in patients achieving pCR compared to those without pCR. The 5-year DFS was approximately 76% in the pCR group versus 53% in the non-pCR group.

The Kaplan–Meier curve comparing 5-year OS between patients achieving pCR and those without pCR in the overall study population is shown in **Supplementary Figure 4**. It demonstrates improved OS in patients achieving pCR compared to those without pCR. The 5-year OS was 82.4% in the pCR group versus 56.3% in the non-pCR group (HR 0.48, 95% CI 0.25–0.92; *p* = 0.02).

## Limitation

The study is underpowered to detect moderate differences. HRs for pCR (1.7–1.8) suggest a potential effect that may not reach statistical significance due to sample size limitations.

## Discussion

Locally advanced HER2-positive breast cancers are usually treated with a neoadjuvant approach. However, owing to the poor biological prognostic characteristics of these tumors, this approach is now also being used in early HER2-positive breast cancers. In patients with LABC, NACT helps reduce tumor volume and results in downstaging, thereby making tumors operable and increasing BCS rates. It also helps eradicate micrometastases. Earlier studies have shown that achieving a pCR with NACT combined with anti-HER2 agents is associated with better EFS and OS. Moreover, pCR status after surgery has become an important decision point for adjuvant treatment in HER2-positive breast cancer (13). Chemotherapy combined with dual anti-HER2 therapy is the standard of care in the neoadjuvant treatment of patients with high-risk HER2-positive breast cancer. However, in patients from low socioeconomic settings, anti-HER2 treatment remains unaffordable, particularly dual anti-HER2 therapy and 1 year of trastuzumab. Many patients can afford only 6 months of treatment or shorter trastuzumab regimens administered every 8 weeks. This study describes the pathological response to neoadjuvant treatment using the FinHer protocol in patients with locally advanced HER2-positive breast cancer, as well as the 5-year survival outcomes in terms of pCR and non-pCR. Several trials have evaluated the efficacy of reduced treatment duration, particularly 6 months of trastuzumab. Among these, the most notable trials are the PHARE, HORG, and PERSEPHONE trials. However, only the PERSEPHONE trial demonstrated non-inferior 4-year DFS rates of 89.4%, compared to 89.85% in patients receiving 12 months of trastuzumab, with an HR of 1.07 (95% CI: 0.93–1.24) (14). Nine-week trastuzumab schedules were evaluated in the SOLD and ShortHER trials; however, neither trial demonstrated non-inferiority. In contrast, 5-year update of the ShortHER trial showed favorable outcomes, with 5-year OS rates of 95.2% in the long-duration trastuzumab arm and 95.0% in the short-duration arm (15).

The updated 10-year analysis of the ShortHER trial by Conte et al. shows that 1-year trastuzumab is the standard treatment for patients with HER2+ early BC as noninferiority cannot be claimed. However, numerically, the differences for the patients at low or intermediate risk (N0/N1–3) is negligible, while patients with N4+ have a clear benefit with 1-year trastuzumab (16).

In a meta-analysis by Earl et al., using individual patient data from non-inferiority trials of reduced duration trastuzumab, comparison of 12 m versus 6 m (7,961 patients), non-inferiority was confirmed with a 2.5% critical margin, for IDFS, DRFS, and OS. The meta-analysis demonstrated that 6 m trastuzumab in early HER2-positive breast cancer is not inferior to 12 m and is an option for patients (17).

Notably, all these trials were conducted in the adjuvant setting, whereas our study focuses on the neoadjuvant setting in locally advanced HER2-positive breast cancer. To the best of our knowledge, there are no studies addressing the pathological response to neoadjuvant treatment using the FinHer protocol in this patient population.

The prognostic impact of pCR on survival across intrinsic breast cancer subtypes remains uncertain. However, pCR is associated with a better prognosis in patients with aggressive breast cancer subtypes, such as HR−, G3, HER2+, and TN (18). In our study, patients with the HER2-enriched subtype achieved higher pCR rates compared to those with the luminal B HER2-positive subtype. Moreover, ER/PR positivity was associated with lower pCR rates. In our cohort, 88% of patients underwent mastectomy despite 30% achieving pCR. As per our institutional practice, decisions regarding surgical intervention, such as MRM or BCS, were made prior to the initiation of NACT based on baseline characteristics. Surgical plans were not modified after NACT based on response assessment. No patients had surgery deferred, as none showed disease progression during NACT.

The neoadjuvant approach in operable breast cancer provides an opportunity to tailor postoperative treatment based on pathological response. Current clinical practice increasingly supports escalation of anti-HER2 therapy in patients who do not achieve pCR following dual anti-HER2 treatment during NACT. Escalation treatment using T-DM1 is a standard option in this scenario. Because of the high cost of innovator anti-HER2 targeted therapy, only 8.41%–35.85% of eligible patients are able to receive it in the Indian setting. Nowadays, there is also an opportunity to use biosimilar trastuzumab in resource-limited settings. With the advent of various trastuzumab biosimilars, there has been a rapid increase in its use for the treatment of HER2-positive breast cancer in clinical practice. However, several barriers remain, including high and variable costs among biosimilars, as well as insufficient evidence on their long-term safety and effectiveness. In resource-limited settings, where affordability of even 1 year of biosimilar trastuzumab remains a challenge, shorter HER2-targeted regimens have a definite role. The cost of trastuzumab in India varies significantly depending on the brand and treatment plan. The lowest-priced biosimilar trastuzumab is available at approximately ₹15,749 (190 USD) per 440-mg vial, whereas the innovator brand costs approximately ₹55,000 (643 USD) per 440-mg vial.

As per Gupta et al., at current prices, 1 year of trastuzumab therapy is not cost-effective in India. Reducing the price of the drug by 35% would make 1-year trastuzumab use cost-effective. With a statistically similar number of QALYs gained, 9 weeks of trastuzumab use has a lower incremental cost; hence, its use for 9 weeks is an efficient option in resource-limited settings in India (19).

As per Indian government policies, several publicly financed health insurance schemes have been implemented in India. The most prominent scheme is PMJAY, the largest tax-funded health insurance program for the poor in India, which also includes cancer treatment in its benefit package. This insurance scheme provides coverage for 9-week trastuzumab treatment for poor patients with HER2/neu-positive breast cancer in India.

In the state of Kerala, several health insurance schemes are available, such as the Karunya Arogya Suraksha Padhati (KASP) for economically disadvantaged populations, which enable beneficiaries to afford the 9-week trastuzumab treatment for HER2/neu-positive breast cancer.

Trastuzumab therapy administered for 1 year is often associated with cardiac events, such as asymptomatic ejection fraction (EF) decline, despite its significant survival benefits. Even patients receiving shorter trastuzumab regimens may experience cardiac events. Although the FinHer protocol is considered relatively cardioprotective, trastuzumab-associated cardiotoxicity—manifesting as an asymptomatic decline in LVEF—has been reported at higher-than-expected rates in real-world settings. In a real-world study by TMA et al., involving 300 patients with breast cancer treated with the FinHer protocol, 71 patients (24%) experienced cardiac toxicity. This included asymptomatic EF decline in 62 patients (21%) and symptomatic LVEF decline in 9 patients (3%) (20). In the present neoadjuvant FinHer study, only two patients experienced symptomatic LVEF decline. Seventeen patients (17%) had documented asymptomatic EF decline, and no long-term cardiac morbidity was observed. Affordability remains a significant concern in low- to middle-income countries. However, cost-effectiveness analyses based on published 5-year follow-up results of the FinHer trial suggest that adjuvant 9-week trastuzumab is likely to be a cost-effective treatment option in the Finnish setting (21).

In the 5-year DFS analysis of the SOLD trial, noninferiority of the 9-week treatment could not be demonstrated, with an HR of 1.39 (two-sided 90% CI, 1.12–1.72). In our study, the 5-year DFS for the total study population was 57.8% ± 5.1%, and the 5-year OS was 61.6% ± 5.0%. When stratified by pCR status, the 5-year OS was 72.6% in the pCR group compared to 56.6% in the non-pCR group; however, this difference was not statistically significant (*p* = 0.156), although numerically higher in the pCR group.

Notably, pCR was not found to be a significant predictor of OS or DFS in this study. This may be due to the inclusion of patients with more advanced disease, such as oligometastatic disease, which could have compromised the results. Additionally, the fact that no non-pCR patient received T-DM1 is highly relevant and may have reduced the survival differences between the pCR and non-pCR groups.

In our study, pCR following NACT was low in both stage III and oligometastatic disease, particularly in the context of ER and PR positivity. Luminal B HER2-positive patients demonstrated lower pCR rates compared with the HER2-enriched subtype. The recurrence rate was relatively high, and the 5-year OS was relatively low compared with published studies, likely due to the inclusion of more advanced-stage patients. The lack of an observed association between pCR and survival should be interpreted with caution. In HER2-positive disease, pCR is consistently associated with improved outcomes, particularly when modern anti-HER2 therapies are used. In this study, several factors may explain the lack of statistical significance, including the inclusion of oligometastatic disease, a limited sample size, the use of short-course trastuzumab, and the absence of post-neoadjuvant escalation (i.e., no T-DM1 in non-pCR patients). The data do not support the idea that pCR lacks prognostic value; rather, we suggest that this cohort is insufficient to reliably demonstrate such an effect. Based on the notion that patients with oligometastatic disease can achieve long-term remission, many such patients receive intensive therapeutic approaches, including metastasectomy and stereotactic body radiotherapy. However, evidence of a survival benefit is derived from studies that are limited by small sample sizes, single-institution data from secondary or tertiary referral centers, lack of adequate control groups, and limited follow-up. A study by Steenbruggen et al. demonstrated that, in a real-world nationwide cohort of 3,447 patients with *de novo* metastatic breast cancer, disease limited to one to three metastases was associated with favorable OS, particularly when local therapy of metastases was applied. This benefit was especially evident in premenopausal or perimenopausal patients without lung metastases (22). A major limitation of the study is that the treatment strategy reflects a historical and resource-constrained setting, with no use of pertuzumab, post-neoadjuvant T-DM1, or dual anti-HER2 therapy. The authors caution that these conclusions should not be generalized to modern treatment standards.

The absence of statistically significant survival differences between patients achieving pCR and those not achieving pCR should be interpreted cautiously. Although the observed HRs suggested a potential survival benefit associated with pCR, the relatively small cohort size and limited number of events likely reduced the statistical power to detect moderate survival differences. As a result, the CIs were wide, and the study may have been underpowered to demonstrate statistically significant survival effects.

## Conclusion

The neoadjuvant FinHer protocol resulted in a pCR rate of 30% in patients with locally advanced HER2-positive breast cancer. ER/PR positivity was associated with decreased pCR rates. Achievement of pCR was associated with a numerically lower risk of death and recurrence; however, these differences did not reach statistical significance. Stage III disease remained independently associated with improved survival outcomes compared with oligometastatic disease.

## Data Availability

The original contributions presented in the study are included in the article/**Supplementary Material**. Further inquiries can be directed to the corresponding author.
