# Supplementary material for: Neoadjuvant FinHer regimen in patients with HER2-positive breast cancer: a retrospective audit
Source: Front Oncol. 2026 May 18;16:1752548. doi: 10.3389/fonc.2026.1752548 (PMC13222843; doi:10.3389/fonc.2026.1752548)

Supplementary Figure3: Five-year DFS for total study population with PCR vs Non-PCR

*Kaplan–Meier curve demonstrating the comparison of five-year disease-free survival between patients achieving pathological complete response (pCR) and those without pCR in the overall study population. It showed improved disease-free survival in patients achieving pCR compared to those without pCR. The five-year DFS was* ***approximately 76% in the pCR group versus 53% in the non-pCR group****.*


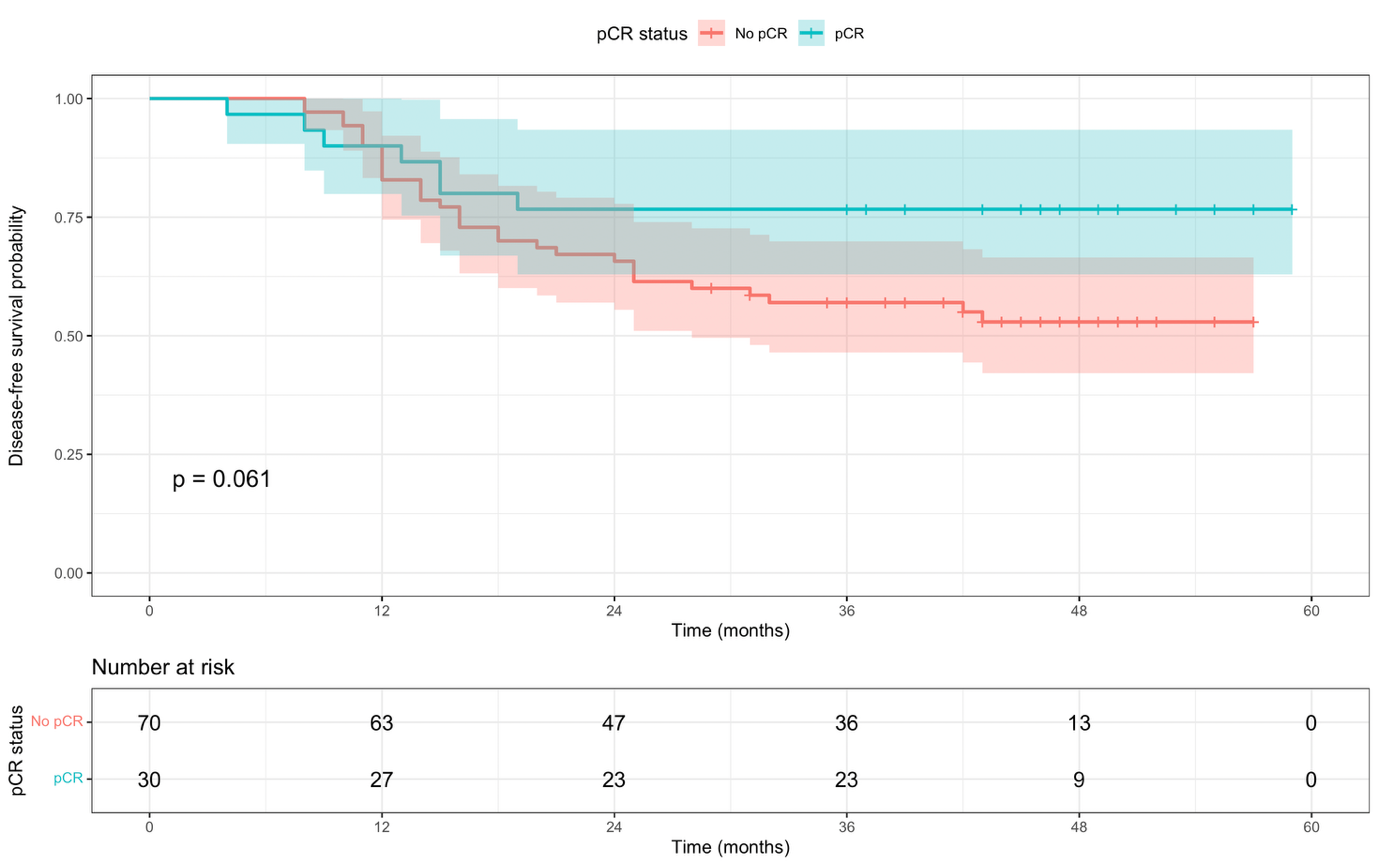


Supplementary Figure 4: Five-year OS for total study population with PCR vs Non-PCR

*Kaplan–Meier curve demonstrating the comparison of five-year overall survival between patients achieving pathological complete response (pCR) and those without pCR in the overall study population.* It showed improved overall survival in patients achieving pCR compared to those without pCR. The five-year OS was **72.6% in the pCR group versus 56.6% in the non-pCR group** (HR 0.48, 95% CI 0.25–0.92; p=0.02).


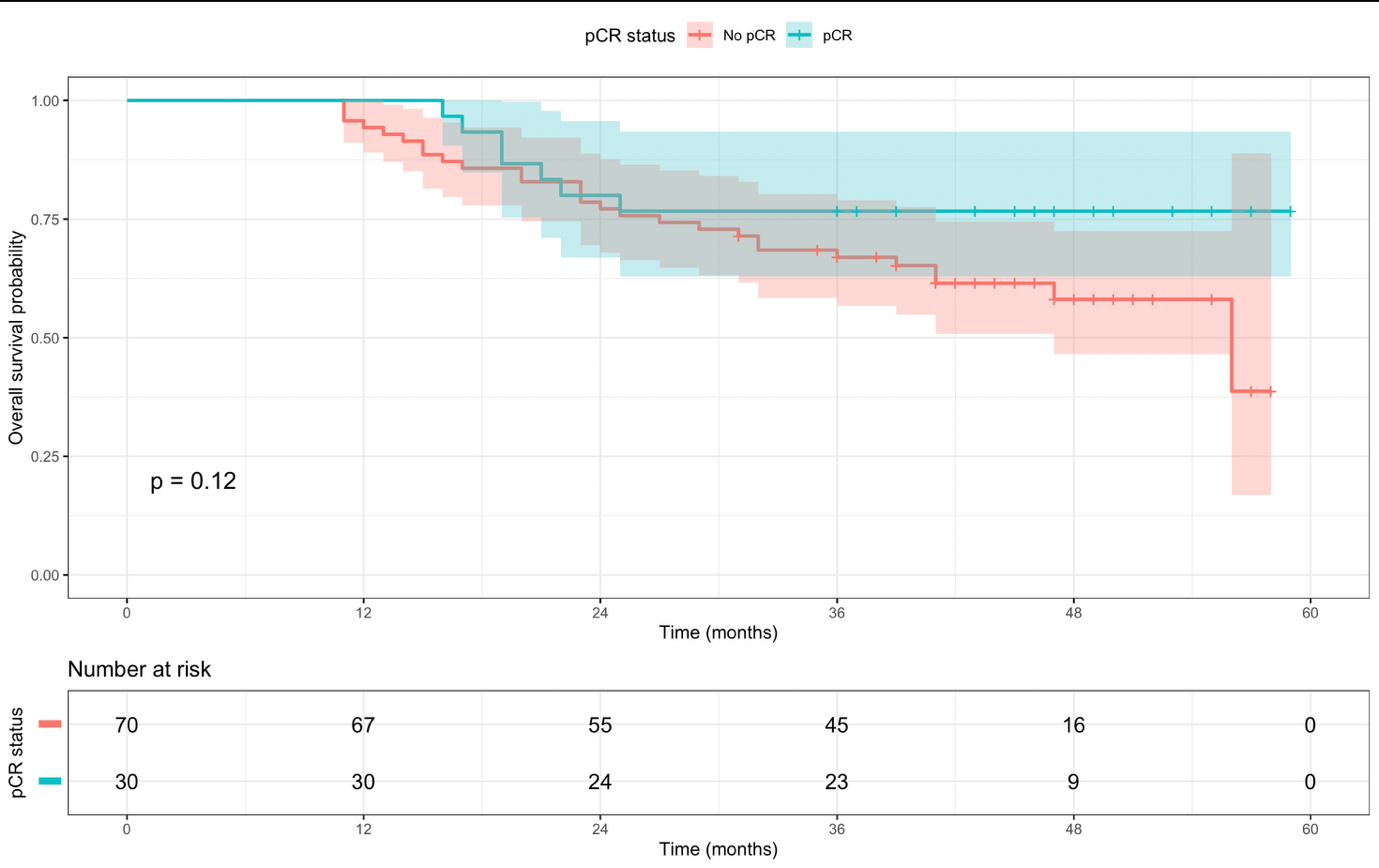

Supplement: Supplementary file 1 [file DataSheet1.docx]
