# Supplementary material for: Neoadjuvant FinHer regimen in patients with HER2-positive breast cancer: a retrospective audit
Source: Front Oncol. 2026 May 18;16:1752548. doi: 10.3389/fonc.2026.1752548 (PMC13222843; doi:10.3389/fonc.2026.1752548)

Supplementary Table I: Five year -Overall Survival in variables

| Variables | 5 years OS (%) | Std error | P-Value |
| --- | --- | --- | --- |
| Age <65  Age >65 | 61.1  65.6 | 5.4  1.4 | 0.742 |
| Pre & perimenopausal  Post menopausal | 54.5  64.8 | 9.2  6 | 0.228 |
| Stage 3  Stage 4 oligomets | 69.9  33.9 | **5.4**  **10.1** | **0.001** |
| Luminal B Her2 positive  Her2 enriched | 62  60.4 | 6.2  8.7 | 0.924 |
| Grade2  Grade3 | 50.2  66.4 | 9.7  5.8 | 0.372 |
| PCR  No PCR | 72.6  56.6 | 8.3  6.2 | 0.156 |
| BCS  MRM | 83.3  58.5 | 10.8  5.4 | 0.122 |

Supplementary Table II: Five year – DFS in variables

| Variables | 5 years DFS (%) | Std error | P-Value |
| --- | --- | --- | --- |
| Age <65  Age >65 | 55.8  72.7 | 5.4  13.4 | 0.205 |
| Pre & perimenopausal  Post menopausal | 46.2  63.4 | 9  6 | 0.05 |
| Stage 3  Stage 4 oligomets | 64.9  34.6 | 5.6  10.1 | **0.002** |
| Luminal B Her2 positive  Her2 enriched | 58.1  56.8 | 6.2  8.9 | 0.814 |
| Grade2  Grade3 | 43.1  64.8 | 9  5.9 | 0.075 |
| PCR  No PCR | 69.2  48.7 | 8.6  7 | 0.117 |
| BCS  MRM | 75  55.4 | 12.5  5.4 | 0476 |

Supplementary Figure 1: Five-year survival in patients with PCR vs Recurrence (DFS)


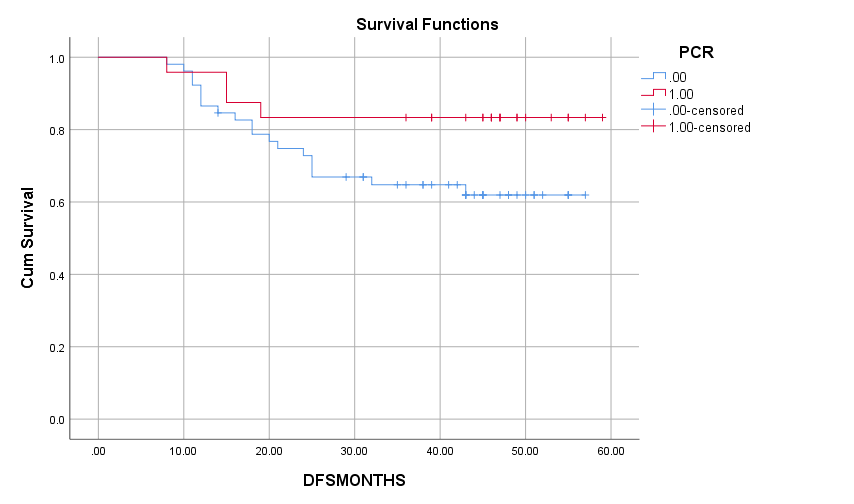


Supplementary Figure 2: Five-year survival in patients with PCR vs Death (OS)


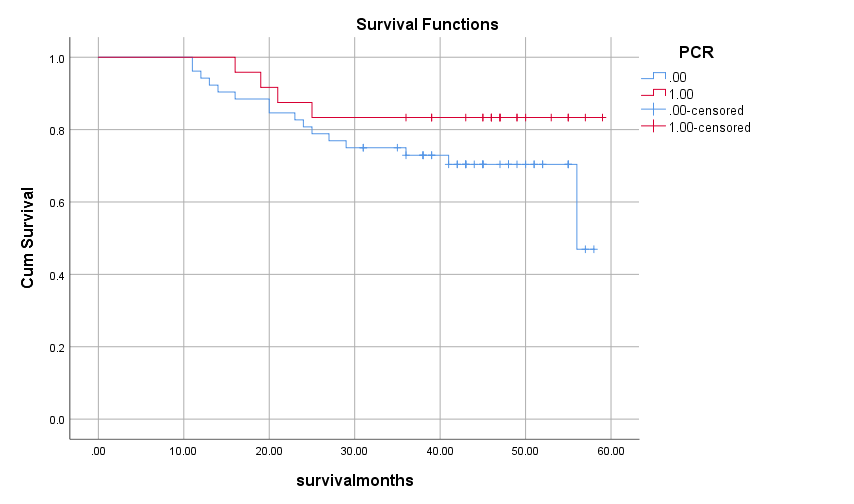

Supplement: Supplementary file 2 [file Table1.docx]
